# Sexuality in schizophrenia: Perception of signals of sexual interest

**DOI:** 10.1192/j.eurpsy.2024.804

**Published:** 2024-08-27

**Authors:** P. Biedková, O. Vaníček, O. Novák, K. Ständer, E. Kolářová, R. Androvičová

**Affiliations:** ^1^1st Faculty of Medicine, Charles University; ^2^Centre for sexual health and interventions, National Institute of Mental Health; ^3^Faculty of Arts, Charles University, Prague, Czech Republic

## Abstract

**Introduction:**

There is emerging evidence that people with schizophrenia (SCH) struggle to form romantic relationships and are often dissatisfied with their sex lives. Intimate relationships are perceived as normalizing and related to a person’s recovery and better medication adherence. Nevertheless, this area remains scientifically unaddressed, and patients with SCH generally do not feel adequately supported in terms of their sexual health.

**Objectives:**

The study aims to assess whether challenges in establishing sexual relationships could be connected to: a) decreased salience of sexual intimacy and/or b) compromised ability to detect, recognize, and react to signals of sexual interest.

**Methods:**

Forty-three patients with SCH (29 males and 14 females) and a control group of twenty-four participants (11 males and 13 females) were exposed to our first experiment, the Circular attention task. This task was designed to evaluate the salience of erotic stimuli compared to neutral ones. At the beginning of each trial, a black fixation circle appeared in the middle of the screen. When a fixation of 250 ms or longer was detected within the circular area of interest (AOI) around the fixation circle, the fixation circle disappeared, and a pair of erotic/neutral pictures appeared. During the experiment, the eye movements were measured using the eye-tracking device Eyelink 1000plus. For data analysis, we used Wilcoxon signed-rank test to assess the differences between the mean latency to first fixation, mean duration of first fixation, and mean proportion of time spent gazing at the stimulus both for sexual and neutral pictures in the whole sample regardless of sex and patient status. More detailed analysis was performed using 2 (sex: male, female) x 2 (status: patient, control) two-way ANOVA.

**Results:**

Considering the whole sample there was a significant difference in mean latency to first fixation (W = 707, p = 0.007, r_rb_ = -0.379) and mean duration of first fixation (W = 1923, p < 0.001, r_rb_ = 0.739). There was a shorter latency to first fixations towards sexual pictures (M = 952.33 ms) than to neutral pictures (M = 1005.30 ms). First fixations were longer for sexual pictures (M = 280.96 ms) than for neutral pictures (M = 243.73 ms). There was an effect overall in the sample towards the sexual pictures, but it was not different for participants based on their sex or patient status.

**Conclusions:**

Findings revealed that interest in explicit sexual stimuli does not differ based on sex or patient status. Patients with SCH appear to find explicit erotic signals sexually salient, suggesting their interest in sexual intimacy. Our study will further investigate whether persons with SCH are able to interpret, recognize and respond to signals of sexual interest. Based on our results, the guidelines for sexological remediation will be developed.

The study was supported by the Charles University, 1. LF project GA UK No. 56123.

**Disclosure of Interest:**

None Declared

